# Competition for water for the food system

**DOI:** 10.1098/rstb.2010.0152

**Published:** 2010-09-27

**Authors:** Kenneth Strzepek, Brent Boehlert

**Affiliations:** 1 University of Colorado, Boulder, CO, USA; 2 The Massachusetts Institute of Technology, Cambridge, MA, USA; 3 Industrial Economics, Inc., Cambridge, MA, USA

**Keywords:** global water resources, water for agriculture, water availability, water demand, climate change and water, environmental flow requirements

## Abstract

Although the global agricultural system will need to provide more food for a growing and wealthier population in decades to come, increasing demands for water and potential impacts of climate change pose threats to food systems. We review the primary threats to agricultural water availability, and model the potential effects of increases in municipal and industrial (M&I) water demands, environmental flow requirements (EFRs) and changing water supplies given climate change. Our models show that, together, these factors cause an 18 per cent reduction in the availability of worldwide water for agriculture by 2050. Meeting EFRs, which can necessitate more than 50 per cent of the mean annual run-off in a basin depending on its hydrograph, presents the single biggest threat to agricultural water availability. Next are increases in M&I demands, which are projected to increase upwards of 200 per cent by 2050 in developing countries with rapidly increasing populations and incomes. Climate change will affect the spatial and temporal distribution of run-off, and thus affect availability from the supply side. The combined effect of these factors can be dramatic in particular hotspots, which include northern Africa, India, China, parts of Europe, the western US and eastern Australia, among others.

## 1. Introduction

Globally, 2600 km^3^ of water are withdrawn each year to irrigate crops, representing over two-thirds of all human withdrawals (FAO 2004). As water scarcity intensifies and many of the world's river basins approach closure (i.e. all water supplies have been put to use for at least part of the year; Smakhtin 2008), water is increasingly transferred out of agriculture to provide for other demands, such as energy generation or growing urban populations. Pimentel *et al*. (1997) note that given worldwide hunger, rising populations will increase pressure on already constrained food supplies. Vorosmarty *et al*. (2000) argue that global water resources are already under stress at current population levels, and that this will only intensify as populations rise further. Perhaps more problematically, rising incomes cause diets to shift to more water-intensive agricultural products and cause levels of water service to increase (e.g. from community standpipes to plumbing systems). Together, these are rapidly increasing *per capita* water demand in developing nations. Simultaneously, to meet higher food demands for a growing population, agriculture is expanding to new regions and becoming more productive, both of which are rapidly increasing the demand for water. Energy consumption and other industrial activities in many countries continue to increase, causing industrial water consumption to rise. Perhaps, the most important and most overlooked, environmental flow requirements (EFRs) are increasingly being recognized as a crucial element of a functioning riparian ecosystem and, accordingly, are increasingly being instated as part of environmental management. As EFRs are instated, remaining water for agriculture will be further diminished. In addition to rising demands on water resources, climate change will significantly affect the timing, distribution and magnitude of water availability. Where shifts in water availability reduce regional water supplies, agriculture may be further threatened.

In *Water for agriculture*: *maintaining food security under growing scarcity*, Rosegrant *et al*. [Bibr RSTB20100152C30][Bibr RSTB20100152C31]) review the recent works on water for agriculture at the global and regional scale. *Water for food, water for life* (Molden 2007) provides a comprehensive review of water management issues in agriculture, and considers how increasing demands and environmental flows could threaten water supplies. However, the analysis considers forecasts of municipal and industrial (M&I) water demands at a broad geographical scale rather than at a more disaggregated national level, and does not quantitatively evaluate how climate change impacts water supply. Strzepek & McCluskey (2007) look at the effects of climate change on agriculture in Africa with water as a primary constraint. This study does not, however, explicitly address whether growing demands and shifting supplies will leave sufficient water for agriculture.

In this paper, we consider the fraction of current agricultural withdrawals that may be threatened given increasing water demands in other sectors, limitations imposed on withdrawals to meet EFRs and the likely effects of climate change. We first briefly review demand- and supply-side factors that will affect water available for agriculture, and then model the possible implications for agricultural water availability through 2050 under climate change. In doing so, we comment on the relative importance of each competing pressure, and identify geographical ‘hotspots’ where water for agriculture could be substantially reduced. Finally, we comment on the most significant sources of uncertainty in our results, and suggest directions for additional research.

## 2. Factors that will affect water for agriculture

### (a) Competing demands

Three of the most significant competing demands for water in agriculture are rising M&I uses (particularly in developing countries) and baseline EFRs. We describe these and others below.

#### (i) Municipal demand

Municipal water demand, as defined here, encompasses both domestic and commercial uses of water. Increases in municipal water use, which will be driven by both rising populations and *per capita* incomes, will vary widely across countries. As noted by Cole (2004) and others, a nation's *per capita* GDP is a strong determinant of its *per capita* municipal water use. As *per capita* incomes rise in poorer nations, level of service moves from systems such as rainwater catchments, truck-supplied water or public standpipes, to plumbing systems where water is delivered directly to households. Gleick (1996) observes that at the lowest levels of service, individuals may only consume an average of 10 litres of water per day, whereas at the highest levels people may consume between 150 and 400 litres per day. The relationship between *per capita* water use and *per capita* GDP growth over time depends on the development path of the particular nation; it is probably that countries with more equitable distributions of resources (i.e. those with lower Gini coefficients) will spread advancements in water service more widely, which will lead to more rapid increases in average *per capita* water use.^1^

Once the majority of a population has ready access to water (as in most developed nations), household and commercial consumption of water flattens with respect to incomes, and then falls with further increases in income as nations introduce or require water-efficiency measures (e.g. water-saving showerheads and toilets). As a result, over the past few decades, nations such as the US and Switzerland have had constant or falling *per capita* municipal water use as *per capita* GDPs have increased (see Kenny *et al.* 2009). This trend has prompted Cole (2004) to inquire whether municipal water use follows an environmental Kuznet's curve, where *per capita* water use initially rises with incomes and then falls as nations grow wealthier. Indeed, as seen in table 1, European water withdrawals generally increased through the 1970s and declined between 1980 and 1995. Given that GDP and population were generally rising through this period, the trend in *per capita* use relative to *per capita* GDP would be considerably lower.

**Table 1.** Trend in total European water withdrawals. Source: Krinner *et al*. (1999).

country	mean annual change in withdrawals (%)		country	mean annual change in withdrawals (%)	
	1970–1980	1980–1995		1970–1980	1980–1995
Austria		0.2	Norway	−1.6	
Belgium	−0.5		Portugal		1
Denmark		−1.9	Spain	5	−1.2
Finland	1.2	−2.9	Sweden	0.1	−2.7
France	4.1	1.1	UK	0.2	−1.5
Germany	2.9	0.8	Estonia		0.5
Greece	5.0		Hungary	4.9	1.9
Italy	3.0	0	Poland	3.4	−1.1
Netherlands	1.1	−1.5			

**Figure 1.**
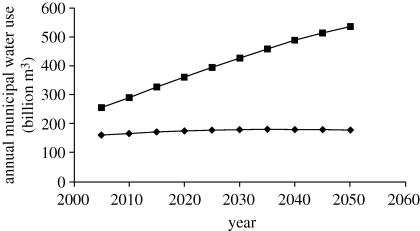
Total projected OECD versus non-OECD municipal water use, 2005–2050. Source: Hughes *et al*. (in press). Squares with solid lines, non-OECD; diamonds with solid lines, OECD.

Developing nations where incomes are rising rapidly, such as China or India, will experience dramatic increases in municipal water use as levels of water service become more advanced. In nations where populations are also rising, these effects will be further magnified. World Bank projections of municipal water use over time for OECD and non-OECD countries are included in figure 1. Note that OECD municipal demand is projected to increase only by 10 per cent (from 162 billion m^3^ to 178 billion m^3^) through to 2050, as compared with the over 100 per cent increase forecast in non-OECD countries (from 257 billion m^3^ to 536 billion m^3^).

#### (ii) Industrial demand

Industrial water demand includes water use for manufacturing, energy generation and other industrial activities. Similar to municipal demand, *per capita* industrial water use tends to rise rapidly as a nation industrializes and then falls as countries move towards more service-based industries. As a result, the most important determinant of future industrial water use is the stage of a country's development. A related factor is whether the country adopts water-conserving technologies. If regulations on water use are imposed that require conservation technologies, or if water prices cause industrial water use to become more costly than conservation, water use will tend to decline. This trend is typified in the construction of new energy generation capacity in developing and developed countries: new power plants in developing countries generally use water for thermoelectric cooling, whereas new facilities in developed nations often use air cooling condensers to avoid excess water use and thermal pollution. In some instances, developed nations transfer lower water use technology to developing nations and thus allow those nations to ‘leapfrog’ past the period during their development paths with highest *per capita* industrial water use.

**Figure 2.**
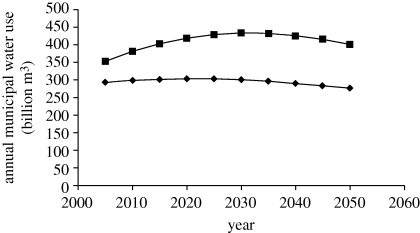
Total projected OECD versus non-OECD industrial water use, 2005–2050. Source: Hughes *et al*. (in press). Squares with solid lines, non-OECD; diamonds with solid lines, OECD.

These patterns can be observed in figure 2, which shows World Bank projections of total OECD and non-OECD industrial water use between 2005 and 2050. Note that total OECD industrial water use declines and non-OECD use increases only slightly after peaking during the 2030s. Industrial water use is dominated by cooling and non-consumptive uses. When faced with pollution controls or high water prices, industrial water use has exhibited major reductions (Kenny *et al.* 2009). The World Bank projections assume that leapfrogging occurs to facilitate reductions in developing nations' industrial use.

#### (iii) Environmental flow requirements

**Figure 3.**
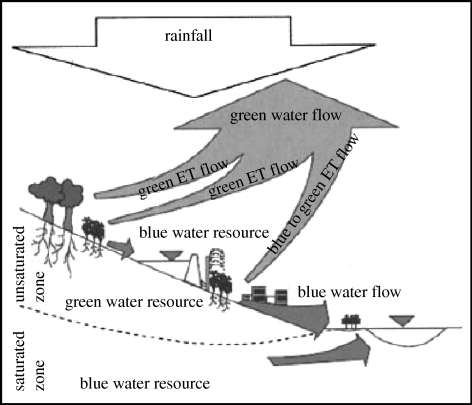
Blue and green water. Source: Falkenmark & Rockström (2006).

EFRs refer to minimum flows allocated for the maintenance of aquatic ecosystem services. EFRs can also be viewed as a demand for floodplain maintenance, fish migration, cycling of organic matter, maintenance of water quality or other ecological services (Smakhtin 2008). Although these demands are increasingly being viewed as crucial, they are often not included in traditional accounting determinations of how close river basins are to closure. In understanding EFRs, Falkenmark & Rockström (2006) differentiate between the ‘blue water’ in lakes, rivers and aquifers that is available for human withdrawal, and the ‘green water’ in soil moisture that is used by terrestrial ecosystems, including agricultural systems (figure 3). They argue that excessive blue water withdrawals can lower water tables and affect the availability of green water, thus potentially impairing terrestrial ecosystem function. Globally, irrigation consumes nearly 1800 km^3^ of blue water annually, with rainfed crops consuming an additional 5000 km^3^ of green water (Falkenmark & Rockström 2006).

**Figure 4.**
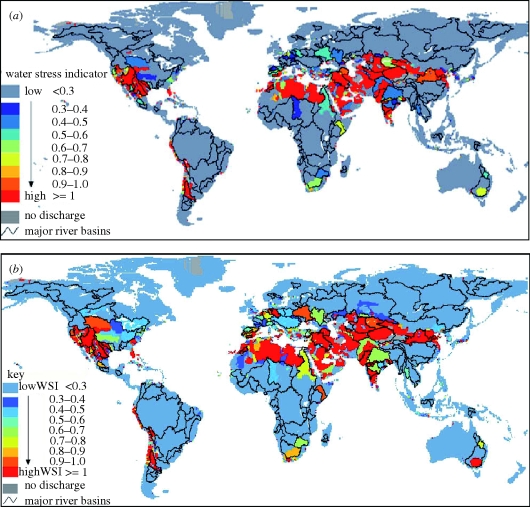
(*a*) Traditional water stress and (*b*) water stress with environmental flows. Source: Smakhtin *et al*. (2004*b*).

As the focus has shifted from maintaining minimum flows to ensuring that the timing and magnitude of flows are appropriate to assure ecosystem health, quantifying EFRs within individual river basins has grown more complex. Smakhtin *et al.* (2004*a*) suggests that Q90 flows (i.e. flows that are exceeded 90% of the time) are sufficient to maintain riparian health in ‘fair’ condition, and are generally a reasonable assessment of EFRs. He contrasts these with the much higher Q50 flows (i.e. flows that are exceeded half the time), which maintain the riparian system in ‘natural’ condition (i.e. negligible modification of habitat) and Q75 flows, which maintain the system in ‘good’ condition (i.e. largely intact biodiversity and habitats despite some development). Depending on the shape of a river's hydrograph, Q90 flows may be exceedingly low (e.g. if greater than 10% of flows are zero, Q90 flows will be zero). In these instances, Smakhtin suggests that high-flow requirements be instated, thereby imposing minimum water flow requirements at the high end of the hydrograph. Figure 4 (from Smakhtin *et al.* (2004*b*)) compares traditional water stress in the world's river basins to water stress with EFRs included. Note the expansion and intensification of stressed basins, particularly in the Middle East, central Asia and southern Europe.

#### (iv) Other considerations

*Increasing agricultural demands*. Food production will need to continue to increase to meet the growing demands of larger, wealthier populations (Tilman *et al*. 2001). At the same time, the increased demand for renewable sources of energy will cause the fraction of land for biofuel production to increase (Fisher & Schrattenholzer 2001; Berndes 2002). To meet these demands, agriculture will move into currently undeveloped lands, which may increase evapotranspiration (ET) if the crops are more water-intensive than the natural vegetation, and will certainly do so if irrigation systems are installed. As incomes and crop prices rise and farmers seek higher yields, sprinkler and flood irrigation systems will be installed in current locations, which will increase both crop water use and evaporation.

*Location of withdrawals*. The relative location of the various demands within the basin is critically important to water availability for agriculture. If M&I demands (described together henceforth) are concentrated upstream of agriculture, water is more likely to remain available for farming because return flows from M&I uses are generally a large percentage of initial withdrawals (roughly 90%). On the other hand, growing cities and industry near the terminus of river basins may transfer water out of upstream agriculture if supplies are constrained, particularly given that ET from agriculture consumes between 50 and 80 per cent of withdrawals, depending on crops grown, climate and irrigation efficiency (Postel *et al*. 1996). EFRs also have a spatial dimension because these flows must remain in rivers throughout their course. This may be an issue in cases where M&I uses withdraw large volumes of water upstream and return the majority downstream, creating river segments with flows that are below EFR targets.

*Political and institutional issues*. Political and institutional issues may also affect availability of water for agriculture. Transboundary competition for water can cause water to be used for domestic agricultural production to maximize local production rather than where regional agricultural productivity (i.e. ‘crop per drop’) is highest. This causes an effective loss of productivity for agriculture. In addition, many countries have national security and economic policies focused on reaching food self-sufficiency. This focus is driving many countries to withdraw water for agriculture in water-stressed basins rather than importing agricultural products. While it may be economically feasible for the nations to import food, the desire not to be held hostage by food exporters can lead to environmentally unsustainable water use.

In addition, the presence or absence of water markets can have a significant effect on the availability and distribution of water for agriculture. In regions where broad water markets exist, such as southeastern Australia or certain parts of the western US, water prices are often driven by demands with higher marginal values than agriculture, such as urban uses. Generally, this has the effect of transferring water out of agriculture to these higher value uses. In other instances, water markets have been successfully established in many regions to transfer water between agricultural products, typically to higher value products (e.g. from alfalfa to fruit trees). The majority of nations currently lack water markets owing to legal or institutional barriers, poor water metering infrastructure and/or exceedingly high transaction costs; however, increasing water scarcity may cause markets to become more prevalent in future years.

### (b) Changing water availability

Next, we discuss the potential effects of climate change and groundwater depletion on availability of water for agriculture.

#### (i) Climate change

Climate change affects the water cycle through changes in temperature, the timing and magnitude of precipitation, soil moisture, run-off, the magnitude and frequency of extreme events, and a number of secondary effects. Although precipitation is often projected to increase under climate change, research has suggested that a 4ºC temperature increase would require at least a 10 per cent increase in precipitation to balance evaporative losses. As a result, in many regions projected increases in precipitation can accompany decreases in run-off (Gleick 2000). Spatial patterns of these changes in run-off will vary widely. For example, models predict that run-off will increase by 10–40% in eastern equatorial Africa and that run-off will decline by 10–30% in southern Africa (Milly *et al*. 2005). In addition, a warmer climate brings with it increases in the magnitude and frequency of extreme events (Bates *et al.* 2008). The magnitude and distribution of run-off will also be further affected by reductions in glacial melt.

Climate change may also have several secondary effects that impact the water cycle. Increases in the intensity of precipitation events, coupled with extended periods of lower streamflow, may intensify pollution issues (Kundzewicz *et al.* 2007). Groundwater systems are anticipated to respond more slowly to changes in climate than surface water systems, but increase in evaporation, changes in vegetation, increases in high run-off events and other effects of climate change may reduce the potential for groundwater infiltration. The net effect of these changes may be reduced sustainable levels of groundwater pumping, changes in water availability in surface water systems, or both. Finally, decreases in precipitation coupled with increasing temperature in certain regions will have a pronounced downward effect on soil moisture (a function of soil type, rainfall patterns and temperature patterns), making less ‘green water’ available for crop use (Cao & Woodward 1998; Falkenmark & Rockström 2006).

On the demand side, climate change will directly affect water use across numerous sectors. On agricultural or other vegetated lands, increasing temperatures will cause plant growth (and thus water demand) to increase as long as soil moisture is not constraining. Increased temperatures also increase domestic demand for water (Goodchild 2007), which will be driven primarily by increased garden and lawn watering (Arnell 1998). Rising temperatures may also directly increase water withdrawals for thermoelectric cooling, and indirectly increase cooling withdrawals as electricity demand increases for air conditioning (Bates *et al.* 2008).

The regional effect of these supply and demand effects on water available to agriculture ranges widely, and the fact that both vulnerability and adaptive capacity to changes in climate also differ across regions will magnify differences in the response to changes in water availability (Adger *et al*. 2003).

#### (ii) Groundwater

Between 1950 and 2000, global groundwater extraction has increased sharply to supply municipal, industrial and agricultural uses. As a result, in many regions of the world, groundwater reserves have declined to the point where well yields have fallen dramatically, land has subsided and aquifer salinization has occurred (Konikow & Kendy 2005). In Yemen, for example, groundwater withdrawals exceed recharge by 400 per cent, which prompted the World Bank to express concerns that groundwater mining in the nation threatens the fundamental wellbeing of its citizens (Shah *et al*. 2000). Because shallow groundwater aquifers and surface water bodies are connected through the same hydrological system, excessive groundwater withdrawals will cause increased groundwater infiltration and thus reduced run-off; for example, in Idaho in the northwestern US, farmers, businesses and cities were ordered to shut down 1300 wells to restore reduced spring discharge (Konikow & Kendy 2005). As a result, groundwater pumping is either from hydrologically disconnected sources that have very low recharge rates (i.e. groundwater mining), or directly decreasing the mean annual run-off (MAR) of a surface water source (Winter *et al.* 1998). As the global demand for groundwater continues to increase, groundwater tables and well yields will decline more rapidly, decreasing surface water run-off and forcing those that rely on groundwater resources to seek new sources. Both will have negative effects on water available for agriculture.

## 3. Modelling methodology

To assess the impacts of changing water demand and supply on water available for agriculture, we model the potential implications of increased M&I withdrawals (considered together), EFRs, and climate change on withdrawals for worldwide agriculture through 2050. Specifically, for a number of geopolitical regions and under three climate change scenarios, we estimate the fraction of current agricultural withdrawals that would be threatened assuming that EFRs and increased M&I demands cause total basin withdrawals to exceed MAR (or total annual withdrawals if they currently exceed MAR because of return flows).^2^ Following Winter *et al.* (1998), we assume that regional groundwater withdrawals deplete river basin run-off and therefore implicitly consider subsurface water in our modelling exercise. It must be noted that this analysis may underestimate threats to agriculture, for two reasons: (i) we make these comparisons relative to current agricultural demands rather than the expected higher demands of 2050; and (ii) we do not consider the effects of drought or increased extreme events. On the other hand, the analysis may overestimate threats because we model withdrawals rather than consumptive use and thus do not account for reuse of return flows.

### (a) Overview of the scenarios analysed

We consider a total of three climate change and three demand scenarios. On the demand side, we consider the effects of 2050 M&I demands alone, EFRs alone and 2050 M&I and EFR demands together. M&I demand projections to 2050 are taken from central World Bank projections for 214 countries (Hughes *et al*. in press). EFRs are assumed to be the Q90 basin flows necessary to maintain riparian ecosystems in ‘fair’ condition, and, following Smakhtin, if Q90 flows are exceedingly low owing to the shape of the basin's hydrograph, we assume minimum high-flow requirements to maintain other key ecosystem services (see Smakhtin *et al.* (2004*a*) for details of this approach).^3^

For the climate change analysis, we evaluate a baseline (i.e. no climate change) scenario, and two climate change scenarios based on the range of available general circulation models (GCMs). Although use of GCM ensemble means—with some acknowledgement of the uncertainty in ensemble outputs—has become standard practice in climate research (Bates *et al.* 2008), probabilistic analysis using the full suite of 22 IPCC GCMs was beyond the scope of this work. As a result, we follow the World Bank's economics of adaptation to climate change (EACC) analysis (World Bank 2009), and model the two climate change scenarios under the A2 SRES scenario using the NCAR and CSIRO GCMs, which the Bank considers to represent generally wetter and drier climate runs, respectively.^4^

In total, we consider nine climate-demand scenarios, each compared with the current baseline. Table 2 provides a key for these nine scenarios in a three-by-three grid.

**Table 2.** Nine climate change and demand scenarios.

climate change scenario	demand scenario		
	2050 M&I	EFRs	2050 M&I and EFRs
no climate change	noCC/M&I	noCC/ EFR	noCC/M&I-EFR
NCAR (wet) climate change	wet/M&I	wet/ EFR	wet/M&I-EFR
CSIRO (dry) climate change	dry/M&I	dry/EFR	dry/M&I-EFR

### (b) Modelling approach and data

We use the CLIRUN II hydrologic model in this analysis (Strzepek *et al*. in preparation), which is the latest model in the ‘Kaczmarek school’ of hydrologic models (Yates 1996) developed specifically for the analysis of the impact of climate change on run-off and extreme events at the annual level. CLIRUN II models run-off in 126 world river basins with climate inputs and soil characteristics averaged over each river basin. The model simulates run-off at a gauged location at the mouth of the catchment, and can run on a daily or monthly time step; for this study, climate and run-off data were available on a monthly basis. Because data on 2000 agricultural and M&I withdrawals are available for 116 economic regions of the world, we intersect the 126 river basins with these economic regions to form 281 food production units (FPUs; see Strzepek & McCluskey (2007) and Rosegrant *et al*. (2009*a*,*b*)), which form the geographical unit of our analysis. For each FPU, our baseline data include current MAR values, 2000 agricultural withdrawals and 2000 M&I withdrawals.

We generate 2050 M&I values by first developing ratios of 2050 to current M&I demands using World Bank projections for the 214 countries. Next, we assign each of the FPUs a 2050 to current demand ratio by translating data from the 214 countries to the FPU scale, and then multiply these ratios by 2000 baseline M&I demands to develop 2050 M&I demands for each FPU. We generate EFRs based on the existing run-off distributions in each of the FPUs. On the supply side, climate change will directly affect the MAR within each of the river basins. To assess these changes through 2050, we use the CLIRUN II hydrologic model to generate changes in MAR in each FPU based on the NCAR (wet) and CSIRO (dry) GCMs.

## 4. Modelled threats to water for agriculture

Below, we first present estimates of the percentage of MAR that is: (i) currently withdrawn for agricultural and M&I purposes; and (ii) needed for EFRs and projected 2050 M&I demands. Then, we present the fraction of current agricultural withdrawals in each of the geopolitical regions that may be threatened under the nine scenarios, and conclude this section with a discussion of our findings.

### (a) Water demands in 2000

**Figure 5.**
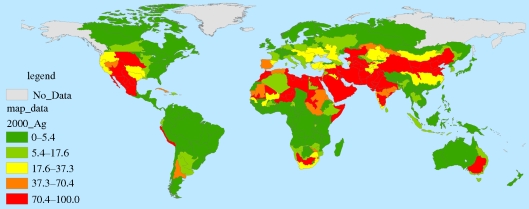
2000 Agricultural water withdrawals as percentage of MAR in 2000.

**Figure 6.**
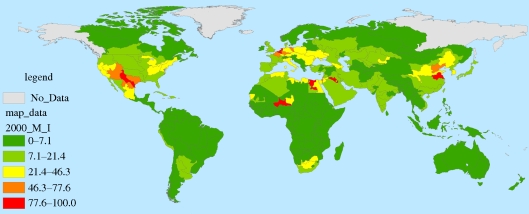
2000 M&I water withdrawals as percentage of MAR in 2000.

Data for the analytical baseline are presented in Table 3 which summarizes the MAR in 2000 for the world and each of the geopolitical regions, along with the percentage of 2000 MAR withdrawn for agriculture and M&I.^5^ In 2000, roughly 10 per cent of worldwide MAR was withdrawn for agriculture and 4.3 per cent was withdrawn for M&I use. Note that in Asia, these figures are 27 per cent and 6.6 per cent, respectively, and in India, agriculture and M&I withdraw 76 per cent and 9.3 per cent, respectively. Figure 5 shows percentage of MAR that is withdrawn for agriculture in 2000. Areas where water is used most intensively for agriculture (e.g. the Middle East, central Asia, western US) are most vulnerable to changes in supply and competing demands. On figure 6, we show the percentage of MAR that is currently withdrawn for M&I—although the magnitude of these values is considerably lower than those of agriculture, these are projected to rise sharply by 2050.

**Table 3.** Total MAR, and agricultural and M&I withdrawals as percentages of MAR for the geopolitical regions.

foresight region	2000 MAR (billion m^3^)	per cent of 2000 MAR	
		2000 agriculture (%)	2000 M&I (%)
**World**	**28 488**	**10.3**	**4.3**
*Europe*	*2871*	*9.2*	*10.7*
European Union	1294	7.3	15.9
Northwestern Europe	739	2.1	15.5
UK	151	0.4	8.4
Former Soviet Union	1701	10.9	7.9
*Africa*	*3882*	*6.4*	*1.1*
Sub-Saharan Africa	3546	1.4	0.4
Nile River Basin	261	56.0	5.8
*North America*	*2521*	*10.1*	*12.4*
*Asia*	*7588*	*27.1*	*6.6*
China	1420	39.3	15.3
India	1140	75.9	9.3
*Latin America and the Caribbean*	*8603*	*2.1*	*1.0*
Brazil	4533	0.5	0.6
*Oceania*	*941*	*5.4*	*0.8*

### (b) Changes in water supply and demand

To evaluate the effects of changing water withdrawal and availability conditions, we model changes in M&I demands, EFRs and changes in run-off caused by a wet and dry climate change scenario through to 2050. For each of the geopolitical regions, table 4 presents the EFR and 2050 M&I withdrawals as percentages of MAR in 2000, and presents percentage changes from MAR under the wet (NCAR) and dry (CSIRO) climate scenarios. Note that regionally, EFRs are between approximately 23 and 54 per cent (Nile River Basin and Oceania, respectively), which are substantial shares of annual flow to satisfy minimum ecological requirements. Between 2000 and 2050, M&I is projected to rise globally from 4.3 to 5.9 per cent of MAR, with the highest rise occurring in India (9.3–24% of MAR). Climate change increases global MAR under both the wet and dry scenarios, although at the regional level the NCAR and CSIRO GCMs projections diverge, sometimes dramatically (e.g. Nile River Basin).

**Table 4.** EFR and 2050 M&I withdrawals as percentages of 2000 MAR and changes in MAR under the 2050 wet (NCAR) and dry (CSIRO) climate scenarios for the geopolitical regions.

foresight region	2000 MAR (billion m^3^)	per cent of 2000 MAR		per cent change from 2000 MAR	
		EFR (%)	2050 M&I (%)	2050 wet climate (%)	2050 dry climate (%)
**World**	**28 488**	**38.7**	**5.9**	**6.4**	**4.7**
*Europe*	*2871*	*45.4*	*10.6*	*9.4*	*8.5*
European Union	1294	48.7	16.3	−3.9	−1.8
Northwestern Europe	739	51.5	15.6	−0.4	8.2
UK	151	31.7	8.9	−6.2	6.0
Former Soviet Union	1701	43.2	8.0	18.7	15.6
*Africa*	*3882*	*33.6*	*2.8*	*1.9*	*−3.4*
Sub-Saharan Africa	3546	34.5	1.6	2.6	−3.3
Nile River Basin	261	23.1	12.2	2.1	−8.4
*North America*	*2521*	*38.5*	*12.0*	*2.7*	*13.3*
*Asia*	*7588*	*33.8*	*11.2*	*3.5*	*8.0*
China	1420	26.5	17.5	2.1	10.9
India	1140	23.3	23.7	7.5	8.1
*Latin America and the Caribbean*	*8603*	*32.9*	*1.6*	*7.3*	*0.7*
Brazil	4533	30.8	0.9	9.2	6.6
*Oceania*	*941*	*54.5*	*0.9*	*12.0*	*0.6*

**Figure 7.**
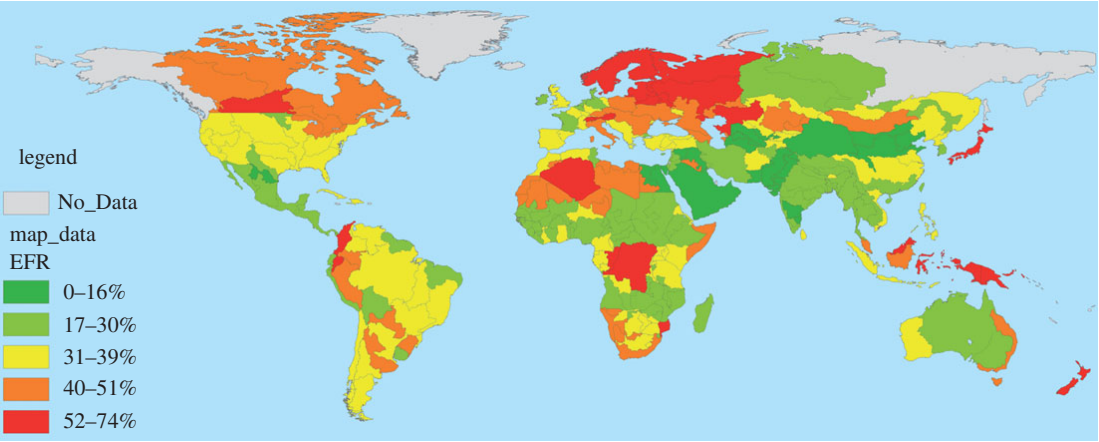
Environmental flow requirements as percentages of MAR in 2000.

**Figure 8.**
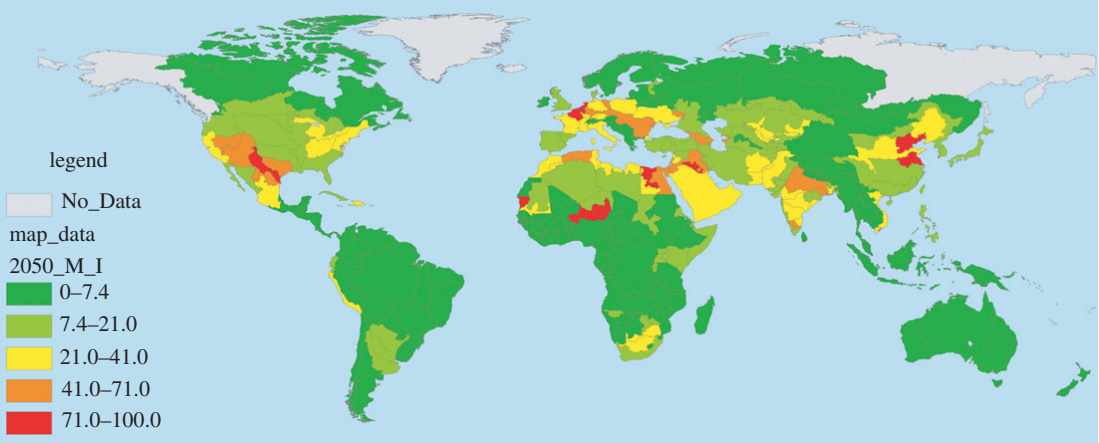
2050 M&I withdrawals as percentages of MAR in 2000.

**Figure 9.**
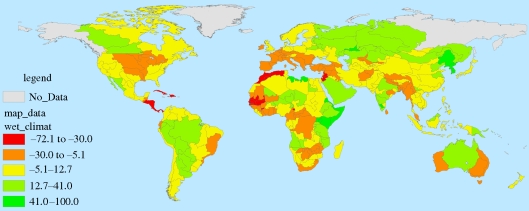
Percentage change in MAR under the wet (NCAR) climate scenario.

**Figure 10.**
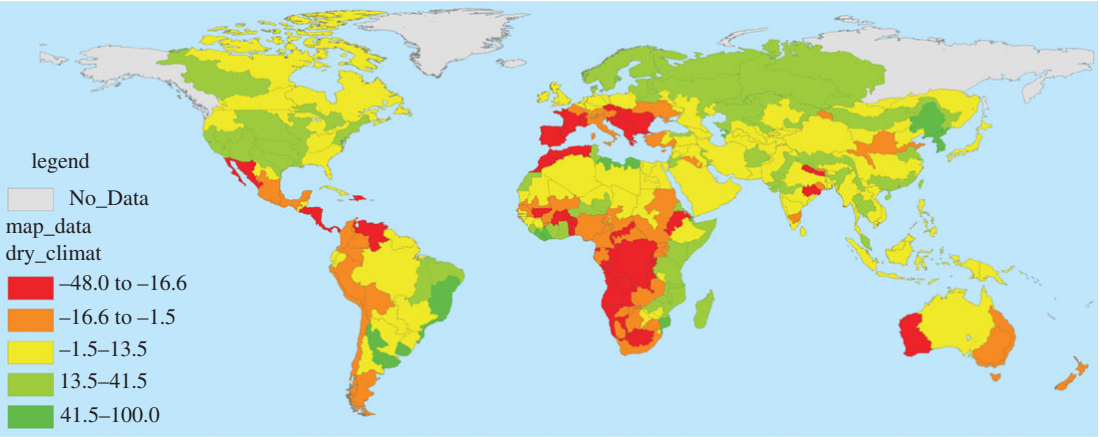
Percentage change in MAR under the dry (CSIRO) climate scenario.

Figures 7–10 present these water demand and climate change estimates spatially for the globe. Note that in certain FPUs, EFRs can be as high as 52–74% of MAR (figure 7), and that 2050 M&I use tends to be highest in areas with higher incomes (figure 8). As can be observed in figures 9 and 10, under climate change, effects on MAR vary widely between the two scenarios and across space.

### (c) Threats to water availability for agriculture

As discussed above, demands for additional M&I withdrawals and minimum EFRs may be met through transfers from agriculture. Table 5 displays the fraction of 2000 agricultural water withdrawals that may be threatened in each of the geopolitical regions under the nine scenarios. Under the no climate change scenario, our models indicate that increases in M&I demands, EFRs, and combined M&I demands and EFRs will require 7.3 per cent, 9.4 per cent and 18 per cent, respectively, of worldwide agricultural water in 2000. Agricultural water in Asia accounts for over two-thirds of the global total, and also accounts for the majority of threatened agricultural water by volume, largely because of substantial increases in M&I demands in India. Modelling indicates that EFRs and M&I increases together will threaten nearly 20 per cent of agricultural water in the European Union and the former Soviet Union. In sub-Saharan Africa, rapidly rising M&I demands also threaten water for agriculture.

**Table 5.** Per cent of agricultural water threatened in the geopolitical regions, nine scenarios.^6^

foresight region	2000 agricultural withdrawals (billion m^3^)	no climate change			NCAR (wet) climate change			CSIRO (dry) climate change		
		2050 M&I (%)	EPRs (%)	2050 M&I and EPRs (%)	2050 M&I (%)	EFRs (%)	2050 M&I and EFRs (%)	2050 M&I (%)	EFRs (%)	2050 M&I and EFRs (%)
**World**	**2946**	**7.3**	**9.4**	**17.7**	**7.1**	**9.1**	**16.5**	**7.0**	**9.1**	**16.9**
*Europe*	*263*	*2.5*	*7.7*	*14.4*	*2.5*	*9.6*	*12.9*	*2.8*	*16.5*	*20.4*
European Union	95	0.7	12.8	18.7	0.7	21.2	19.0	1.6	39.0	37.0
Northwestern Europe	16	4.5	11.7	8.2	4.5	14.6	10.2	3.2	10.4	8.2
UK	0.6	0	0	0	0	0	0	0	0	0
Former Soviet Union	186	3.2	10.0	19.7	3.2	11.4	17.4	3.7	12.3	18.9
*Africa*	*246*	*9.8*	*5.8*	*15.8*	*10.4*	*6.8*	*16.9*	*10.4*	*6.6*	*16.9*
Sub-Saharan Africa	50	11.9	7.2	16.4	11.9	7.7	17.6	12.1	7.3	16.6
Nile River Basin	146	9.1	0.2	9.2	9.1	0.2	9.2	9.1	0.2	9.6
*North America*	*255*	*−0.1*	*15.2*	*14.9*	*−0.1*	*13.8*	*13.6*	*−0.1*	*12.0*	*12.0*
*Asia*	*2060*	*8.8*	*8.9*	*18.6*	*8.6*	*7.8*	*16.7*	*8.3*	*7.4*	*16.8*
China	558	2.7	7.3	10.1	2.3	4.5	6.9	2.3	4.5	6.9
India	866	13.5	12.1	27.7	13.1	11.5	25.5	12.5	10.7	25.7
*Latin America and the Caribbean*	*182*	*3.8*	*12.3*	*16.1*	*4.4*	*15.7*	*19.9*	*3.8*	*12.3*	*16.8*
Brazil	21	0	0	0	0	0	0	0	0	0
*Oceania*	*50*	*0.2*	*14.3*	*14.5*	*0.2*	*14.3*	*14.5*	*0.2*	*14.3*	*14.5*

Under climate change, threats to agricultural water both increase and decrease, depending on the region and scenario. In Europe, less water for agriculture is threatened under the wet scenario, and significantly more is threatened in the dry scenario. We project that threats decline in North America and Asia under both climate scenarios, but increase in Africa and Latin America and the Caribbean.

Note that not all areas will be affected; model results indicate that agricultural water in Brazil and the UK, both of which have plentiful supplies relative to demands (see tables 3 and 4), will not be threatened under any of the scenarios.

**Figure 11.**
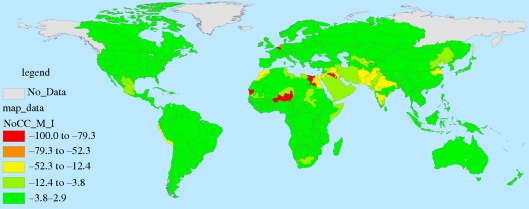
Per cent of agricultural water threatened under the no climate change scenario, given 2050 M&I withdrawals.

**Figure 12.**
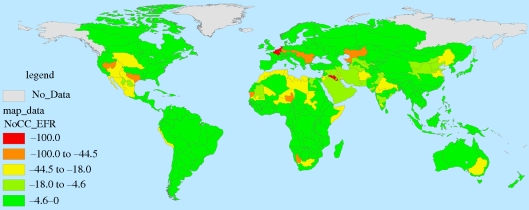
Per cent of agricultural water threatened under the no climate change scenario, given EFRs.

**Figure 13.**
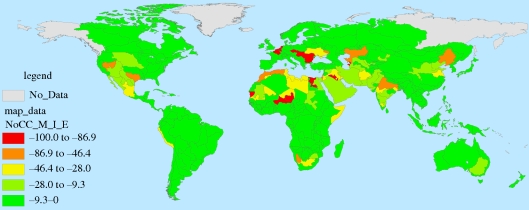
Per cent of agricultural water threatened under the no climate change scenario, given 2050 M&I withdrawals and EFRs.

**Figure 14.**
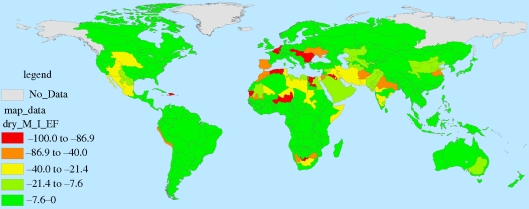
Per cent of agricultural water threatened under the dry (CSIRO) climate change scenario, Given 2050 M&I withdrawals and EFRs.

These results are presented spatially for FPUs in figures 11–14. These spatial representations allow us to identify hotspots where agricultural water will be most threatened. Threats to agricultural water availability given 2050 M&I demands, EFRs and the two combined are presented in figures 11–13, respectively. Figure 14 presents the effects of combined 2050 M&I demands and EFRs under the dry (CSIRO) climate scenario.

In the no climate change scenario, increases in M&I demands tend to affect areas with both high water stress and rapidly growing water demands, explaining why these impacts are concentrated in developing countries. Imposing EFRs, on the other hand, would reduce water supplies in basins with high water stress in both developing and developed countries (e.g. the Colorado River Basin in the US, parts of the Nile River Basin, the Murray-Darling Basin in Australia). Taken together, these increases in demand are most significant in parts of Europe, southern Asia, northern Africa and the western US. As observed above, climate change affects the distribution of water availability, increasing threats to agriculture in some areas and lessening them in others. The shifting locations of hotspots under the dry climate change scenario can be observed on figure 14.

### (d) Discussion

The above results indicate that increasing M&I water use and EFRs will pose significant threats to agricultural water availability. Here, we discuss possible solutions to ensure that agriculture and other demands are satisfied and how to address uncertainties that exist in both climate and water demand projections.

#### (i) Possible solutions

Many alternatives are available to extend limited supplies of water resources, generally falling into the categories of demand management or supply augmentation. Demand management approaches involve using mechanisms to reduce demand such that existing supplies can be extended. For example, Postel (1998) finds that improving the water productivity of agriculture will be critical to meeting future food demands. As water productivity (i.e. irrigation efficiency) increases, agricultural water withdrawals decrease, although consumptive use remains constant. Water conservation in cities or sharing of water-saving technologies with developing countries may be functional approaches to reduce M&I withdrawals and therefore relieve pressure on agriculture. On the supply augmentation side, desalination may be an increasingly realistic alternative as the technology becomes cheaper, and importing of virtual water (Allan 1998) in the form of food and other water-intensive goods can expand supplies and transfer water from water-rich regions to water poorer nations. Hoekstra & Hung (2005) find that 13 per cent of the water used for crop production globally is used for export instead of domestic consumption. Other frequently proposed solutions to water availability issues are water banks and markets. Research in economics has long demonstrated the efficiency benefits from water trading (e.g. Howe *et al*. 1986); however, such efficiency gains tend to transfer water away from agriculture to uses with higher marginal economic values.

#### (ii) Uncertainty

Projections of future water use and availability are highly uncertain owing to underlying uncertainties in their determinants (e.g. GDP projections, variability in climate models). Currently, several studies are developing or have developed probability distributions for these uncertain variables. For example, the International Institute for Applied Systems Analysis (IIASA) has developed population projection fractiles for the world, as described in another Foresight Global Food and Farming Futures Project paper in this volume (Lutz & Samir 2010). These fractiles provide uncertainty bounds on population that are year-dependent. In an ongoing study, the Massachusetts Institute of Technology (MIT) has used Latin-hypercube sampling to develop a joint probability density function (PDF) that captures ranges of the determinants of climate change. When this PDF is complete, climate change analysts will be able to sample directly from this distribution to develop probabilistic estimates of economic and physical climate change effects. In the context of this study, such a PDF would enable a statistical treatment of population, GDP, and other variables that determine future M&I water use.

## 5. Conclusions and extensions

### (a) Summary and conclusions

In this paper, we review the primary threats to agricultural water availability, and model the potential effects of increases in M&I water demands to 2050, EFRs, and changing water supplies given climate change to 2050. For each FPU, we assume that the MAR is the maximum quantity that can be withdrawn annually (or total current withdrawals if they exceed MAR), and that any withdrawals exceeding this limit may come from agriculture. We find that EFRs and increased M&I water demands together cause an 18 per cent reduction in the availability of worldwide water for agriculture by 2050. Meeting EFRs, which can necessitate more than 50 per cent of the MAR in a basin depending on its hydrograph, presents the single biggest threat to agricultural water availability. Next are increases in M&I demands, which are projected to increase upwards of 200 per cent by 2050 in developing countries with rapidly increasing populations and incomes. The combined effect of these increasing demands can be dramatic in key hotspots, which include northern Africa, India, China, parts of Europe, the western US and eastern Australia, among others. These areas tend to be already water-stressed owing to low water supplies, existing large-scale agricultural or M&I demands, or both.

Climate change will affect the spatial and temporal distribution of run-off, and thus change availability from the supply side. Based on wet and dry climate scenarios, we find that water availability for agriculture increases in North America and Asia, and decreases in Africa and Latin America and the Caribbean. In Europe, water availability increases under the wet model and decreases under the dry model. Overall, however, our results indicate that climate change is a much smaller threat to agriculture than growing M&I demands and EFRs.

### (b) Extensions

We suggest two avenues for further research. First, a more rigorous modelling effort of water availability for agriculture based on a more detailed quantification of changes in competing water uses and in availability, as well as a range of GCM outputs and SRES scenarios. Importantly, conduct a sensitivity analysis on results using the joint PDF of climate drivers from MIT's latin hypercube sampling. Second, investigate the causes of increased domestic water demand in different economies, focusing on the relationship with water availability *per capita*, urbanization, income distribution and levels of service (e.g. private delivery, community standpipe, etc). Although rising domestic water use will be one of the main causes of increased global demand for water, existing projections of domestic use have ignored some of these crucial factors.

## Endnotes

1 The Gini coefficient is a measure of income inequity—the higher the coefficient, the less equitable the distribution of incomes in the country.

2 In this paper, our focal ‘geopolitical regions’ are Europe, Africa, North America, Asia, Latin America and the Caribbean, and Oceania. Within Europe, we also focus on the European Union, northwestern Europe, UK and the former Soviet Union. Sub-Saharan Africa and the Nile River Basin are reported for Africa, and in Asia, we report findings for India and China. Finally, we identify impacts on Brazil.

3 Note that the analysis assumes that those basins that currently do not meet Q90 flows today will do so in the future.

4 In the A2 scenario, population growth increases throughout the twenty-first century, but economic growth is regional and occurs more slowly than in the A1B and A1 scenarios. As a result, emissions are lower in 2050 than in the other A storyline scenarios. Note that the SRES scenarios developed in 2000 assume emissions projections that are far more optimistic than has been observed in the past decade (for more detail, see IPCC 2009).

5 Note that the ‘Europe’ Foresight region was listed as containing the former Soviet Union. As a result, we have included all of the former Soviet Union countries in the Europe region, even though many of these are also in Asia.

6 Note that agricultural water availability in North America increases by 0.1 per cent under the 2050 M&I scenarios. This occurs because 2000 M&I and agricultural withdrawals in North America exceed MAR in the Colorado and Rio Grande Basins, but M&I declines in 2050. As a result, additional water is made available to these constrained basins.
